# Sex Differences in Stroke Risk Factors and Mechanisms in a Multi-Ethnic Asian Population

**DOI:** 10.3390/jcdd12080304

**Published:** 2025-08-12

**Authors:** Narayanaswamy Venketasubramanian

**Affiliations:** Raffles Neuroscience Centre, Raffles Hospital, Singapore 188770, Singapore; ramani_nv@rafflesmedical.com; Tel.: +65-6311-1111

**Keywords:** stroke, ischaemic stroke, haemorrhagic stroke, subarachnoid haemorrhage, sex, differences, risk factors, Asian population

## Abstract

Introduction: Previous studies have reported sex differences in stroke. There are few Asian studies. This study was performed to investigate sex differences in stroke risk factors and mechanisms in a multi-ethnic Asian population. Methods: Data on patients admitted to Raffles Hospital for stroke were analysed. Data were extracted on sex, age, hypertension, diabetes mellitus (DM), hyperlipidaemia, smoking, heart disease, and prior cerebrovascular events (pCeVD). Stroke was subtyped into haemorrhagic stroke (HS) or ischaemic stroke (IS) based on brain scan. IS mechanism was categorised using Trial of Org 10172 in Acute Stroke Treatment (TOAST) classification, while the clinical syndrome by Oxfordshire Community Stroke Project (OCSP) classification. Results: Data were collected on 1165 patients, mean age 65.6 ± 12.9 yr; 47.4% female, 83.0% Chinese, with hypertension (63.5%) and hyperlipidaemia (60.3%) being the most common risk factors. HS comprised 23.5%. On regression analysis, compared to males, females had older age (OR 1.03, 95%CI 1.02–10.4) and DM (OR 1.60, 95%CI 1.11–2.30), but less smoking (OR 0.09, 95%CI 0.07–0.13), pCeVD (OR 0.67, 95%CI 0.49–0.93), and HS (OR 0.71, 95%CI 0.51–0.98). There were no differences in HS mechanisms, or IS mechanisms or syndromes. Sex–ethnic differences were found (*p* < 0.001), with more Chinese and fewer Indians among females compared to males. Conclusions: This study corroborates previous studies of significantly older age and more diabetes mellitus, but less smoking and haemorrhagic stroke among female stroke patients compared to males; differences in HS and IS mechanisms were not found. Novel in this study is that sex–ethnicity differences were found. Future studies should prospectively validate these sex/ethnic differences.

## 1. Introduction

Stroke is a major cause of death and disability globally [1]. Previous reviews have reported sex differences in stroke epidemiology [2,3,4]. A meta-analysis of 59 incidence studies (n = 30,404) showed that the mean age at first-ever stroke was 72.9 years among females and 68.6 years among males, with the incidence rates of subarachnoid haemorrhage (SAH) being higher among females, and of brain infarction and intracerebral haemorrhage (ICH) being higher among males [5]. Data from the Global Burden of Disease 2021 study that showed that age-standardised incidence of ischaemic stroke (IS) and SAH were similar between females and males but haemorrhagic stroke (HS) was lower among females [6]. This was corroborated by a meta-analysis of 70 population-based studies that included 19,470 ICH patients from 26 different countries, with 62 studies on crude incidence—the incidence of ICH was lower in females than in males [7].

Risk factors also differ between females and males. In a meta-analysis of 45 articles (n = 673,935), globally, among IS, females were older, suffered more hypertension and atrial fibrillation, but were less likely to have diabetes mellitus or hyperlipidaemia, or drink alcohol or smoke, compared to males [8]. Females also suffered more cardioembolic strokes, while males had more atherothrombotic strokes. A meta-analysis of 42 articles on carotid atherosclerosis found that females more frequently had smaller plaques, and had plaques with calcifications, lipid-rich necrotic core, intraplaque haemorrhage, or an ulcerated plaque less often [9]. A meta-analysis of individual participant data from eight population-based stroke incidence studies with National Institutes of Health Stroke Scale assessment (n = 6343) showed that females had more severe IS but not HS compared to males [10]. There are also unique risk factors found only in females such as hormone therapy, endometriosis, age at menopause, hysterectomy, and oophorectomy [11]. While both females have a higher risk of stroke is they were depressed compared to those without depression, stroke mortality is lower among females with depression than males with depression [12].

There have been reports in recently performed meta-analyses and systematic reviews of sex differences in receiving treatments, post-intervention outcomes, case fatality, return to work, bleeding risks with anticoagulants, perioperative complications after interventions for carotid disease, and treatment responses in neuroprotectant trials [13,14,15,16,17,18,19,20,21,22,23,24] (Table 1). In general, females fared worse than males.

Despite this wealth of information on sex differences in stroke patients, however, there have been few Asian studies [26], and even less so in mixed ethnic populations. This knowledge gap needs to be filled so as to better understand sex differences in stroke globally and explore if sex differences in stroke epidemiology seen elsewhere were also seen among Asians. If there are differences, steps need to be taken determine the extent of and reasons for these differences, and to plan and institute ethnic-specific interventions where appropriate to mitigate these risks.

This study was thus performed to investigate sex differences in stroke risk factors, subtypes and mechanisms in a multi-ethnic Asian population hospitalised for acute stroke in Singapore.

## 2. Materials and Methods

### 2.1. Study Setting

Singapore is an island city-state of 6.04 million people, with a female–male ratio of 1:0.948 [27]. Approximately 75.9% of the citizens and permanent resident visa holders are ethnic Chinese, 15.0% ethnic Malay, and 7.5% ethnic Indians, with the remaining 1.6% being members of “Other” races, which are largely Eurasians. Publicly funded affordable healthcare is widely available [28] at the primary and hospital level, including at the acute care hospitals, step-down community-care facilities and some nursing homes. Private healthcare and healthcare insurance are available. Raffles Hospital is a multi-specialty acute care hospital catering to both private and public patients [29].

The methods of the Raffles Hospital Stroke Study have been previously described [30]. They are explained here below in brief:

### 2.2. Inclusion Criteria

Data on patients who were diagnosed as having an acute stroke were entered into a database if they met the following study criteria: 1. Admitted between 1 June 2015 and 31 December 2023. 2. Clinical diagnosis of stroke based on the World Health Organisation definition of rapidly developing clinical signs of focal (at times global) disturbance of cerebral function lasting more than 24 h or leading to death with no apparent cause other than that of vascular origin [31]. Stroke was also diagnosed if there were global or focal neurological symptoms lasting < 24 h but there was imaging evidence of brain tissue injury attributable to a vascular cause [32]. 3. Admitted within 7 days of onset of stroke symptoms. 4. Had undergone brain imaging by either Computed Tomography (CT) or Magnetic Resonance Imaging (MRI). Patients were excluded if their admission was outside of the study period, stroke had occurred more than 7 days before hospital admission, or if there was no brain scan. They were also excluded if they were not Singapore citizens or permanent residents.

### 2.3. Data Collected

Data were collected on patient age (in years) at time of onset of stroke, sex (female, male—as recorded in the patient’s National Registration Identity Card NRIC), ethnicity (Chinese, Malay, Indian, Others as recorded in the patient’s NRIC), history of vascular risk factors, results of brain imaging to determine stroke subtype, mechanism of IS based on results of investigations, and IS syndrome based on neurological assessment. These data are routinely collected in our stroke pathways.

### 2.4. Risk Factors

Hypertension (HT) was diagnosed if there was a history of hypertension or the patient was ever prescribed medication to lower blood pressure. Diabetes mellitus (DM) was diagnosed if there was a history of diabetes mellitus or the patient was ever prescribed medications to lower blood glucose. Hyperlipidaemia (HL) was diagnosed if there was a history of hyperlipidaemia or the patient was ever prescribed medications to lower blood lipid levels. Smoking was diagnosed if there was a history of ever having smoked a cigarette. Ischaemic heart disease (IHD) was diagnosed if there was a history of ischaemic heart disease or the patient was ever prescribed medications or had undergone interventions for IHD. Previous cerebrovascular disease (pCeVD) was diagnosed if there was a history stroke or transient ischaemic attack or ever prescribed medications or underwent interventions for a previous cerebrovascular event. Female patients were asked for a history of intake of oral contraceptive pills (OCP) or hormone replacement therapy (HRT).

### 2.5. Brain Imaging, Stroke Subtype

All patients underwent CT or MR imaging of the brain to determine stroke subtype [32]. IS was diagnosed if there was infarction of the brain in a defined vascular distribution attributable to ischemia. HS was diagnosed if there was focal collection of blood within the brain parenchyma, ventricular system, or subarachnoid space that was not due to trauma—ICH was diagnosed if there was collection of blood largely within the parenchyma of the brain or within the ventricular system, SAH was diagnosed if the bleeding was largely in the subarachnoid space.

### 2.6. Investigations

The following investigations are routinely performed in all suspected stroke patients—full blood count, kidney panel, glucose and electrocardiogram (ECG). Those with ICH or SAH would also have tests on their prothrombin time (PT) and activated partial thromboplastin time (aPTT). Those with IS would be tested for fasting lipids, transthoracic echocardiography and cardiac monitoring for arrhythmias for up to 72 h. Imaging of the intracranial and extracranial arteries was performed using CT angiogram, MR angiogram, or Triplex ultrasonography.

### 2.7. Ischaemic Stroke Syndrome Classification and Mechanism

The mechanism of IS was assigned using the Trial of Org 10172 in Acute Stroke Treatment (TOAST) classification [33]. Large-Artery Atherosclerosis (LAA was diagnosed if there were clinical and brain imaging findings of either significant stenosis (>50%) or total occlusion of a major brain artery or branch cortical artery, presumed to be due to atherosclerosis. Cardioembolism (CE) was diagnosed if the arterial occlusion was presumed to be due to an embolus arising in the heart. Small-Artery Occlusion (SAO) was diagnosed if the patient had one of the traditional clinical lacunar syndromes and consistent brain imaging. Stroke of Other Determined Etiology (SODE) was diagnosed if there was a rare cause of the stroke such as non-atherosclerotic vasculopathy, hypercoagulable state, or a haematologic disorder. Stroke of Undetermined Etiology (SUE) was diagnosed if the cause of the stroke could not be determined despite extensive investigation, two or more potential causes of stroke were found, or evaluation was incomplete.

The clinical syndrome of IS was classified according to the Oxfordshire Community Stroke Project (OCSP) [34]—lacunar infarct (LACI) if it was a pure motor stroke, pure sensory stroke, sensorimotor stroke, or ataxic hemiparesis; total anterior circulation infarct (TACI) if there was higher cerebral dysfunction, e.g., dysphasia, dyscalculia, visuospatial disorder; homonymous visual field defect; ipsilateral motor and/or sensory deficit of at least two areas affecting the face, arm and leg); partial anterior circulation infarct (PACI) if only two of the three components of the TACI syndrome were present, higher cerebral dysfunction alone, or a motor/sensory deficit that more restricted than for LACI; posterior circulation infarct (POCI) if there was ipsilateral cranial nerve palsy with contralateral motor and/or sensory signs; bilateral motor and/or sensory signs; disorder of conjugate eye movements; cerebellar dysfunction without ipsilateral long-tract signs; and isolated homonymous visual field defect.

All stroke patients were managed by the doctor in charge in consultation with the patient and family. All stroke patients were admitted under the care of one of the 3 neurologists (for IS) or the neurosurgeon (for ICH, SAH) employed by Raffles Hospital.

### 2.8. Study Processes

Patients with a diagnosis of stroke were identified by the Information Technology department of Raffles Hospital, using the International Classification of Diseases, Tenth Revision, Clinical Modification (ICD-10-CM) codes I60–I69, and who had admission dates between 1 June 2015 and 31 December 2023. The case records were reviewed. If the inclusion criteria were met, to respect patient privacy, only non-identifiable data required for the study were recorded, using standardised forms. Data were entered into a database, for subsequent analysis.

### 2.9. Statistical Analysis

Normally distributed continuous data were summarily described by means and standard deviations, non-normally distributed data by medians, while proportions were used for categorical data. To test for associations, unpaired *t*-test was used for normally distributed continuous variables and X^2^ for categorical variables. Variables were then entered into a multivariable logistic regression, with sex as the dependant variable—odds ratios (OR) with 95% confidence intervals (95% CI) were calculated. Associations with *p* < 0.05 were considered statistically significant. Data were analysed using Statistical Package for Social Studies (SPSS) v21.

### 2.10. Ethics

The study was performed in accordance with the Declaration of Helsinki. The study was approved by the Ethics Committee of Raffles Hospital.

## 3. Results

Data were collected on 1165 patients, mean age 65.6 (± 12.9) yr, 47.4% female, mostly Chinese (83%) with Malay (8.6%) and Indian (7.4%) minorities. The most frequent vascular risk factors were HT (63.4%) and HL (60.3%), with SM (35.6%) and DM (31.8%) being among the less common; 24.2% had pCeVD (Table 2). Two females had taken OCP or HRT.

On univariable analysis, females were significantly older and had more DM, but less HL, SM, and pCeVD compared to males; these associations remained significant on multivariable regression analysis except for HL (Table 2). There were also more Chinese and fewer Indians among the females compared to males.

Females had similar HS to males on univariable analysis, but significantly less HS on multivariable regression analysis that included stroke risk factors (OR = 0.71, 95%CI 0.51–0.98) (Table 2).

When only HS was analysed, on univariable analysis, the only significant difference in risk factors was that females smoked less than males; no interethnic differences were found (Table 3). On multivariable analysis of risk factors, however, females were significantly older, had more HT and DM but less HL and SM. There were no differences in the various mechanisms of HS (Table 2). On further regression analysis after adding HS mechanism, age, HPT, DM, HL, and SM remained significantly different. There was no effect of ethnicity.

When only IS was analysed, on univariable analysis, females were significantly older, had more DM and HL, but less SM and pCeVD (Table 4). On multivariable analysis, females remained significantly older, had more HL and less SM and pCeVD. Interethnic differences were again seen, with more Chinese and fewer Indians among the females. There were sex differences in mechanisms of IS on univariable analysis that became insignificant on multivariable analysis; there were no differences in stroke syndromes (Table 4).

## 4. Discussion

This study found that compared to males, female acute stroke patients were older, had more diabetes mellitus, and less smoking and prior cerebrovascular events; there was no significant difference in hypertension, hyperlipidaemia, or ischaemic heart disease. There were also less haemorrhagic stroke patients among females. Among haemorrhagic stroke patients, females were significantly older, had more hypertension and diabetes mellitus but less hyperlipidaemia and smoking; there were no differences in haemorrhage mechanisms. Among ischaemic stroke patients, females were significantly older, had more hyperlipidaemia and less smoking and prior cerebrovascular events, but no sex differences in ischaemic mechanisms or stroke syndromes.

This study showed that fewer females than males were admitted for stroke. While this may be due fewer females living in the vicinity of the study hospital or fewer females accessing acute stroke care, a lower proportion has also been reported in Bangladesh (41.8%) [35], Hong Kong (46.7%) [36], Japan (42.1%) [37], Sri Lanka (40.8%) [38], and Vietnam (38.7%) [39]). The other likely explanation is the lower stroke incidence in females compared to males [5] that may be due to a lower frequency of vascular risk factors such as SM [40], and the vascular protective effect in females of endogenous oestrogens on the endothelia, promoting vasodilation, blood flow, vasoreactivity, anti-inflammatory effects that might be modulated by antioxidant and anti-apoptosis effects [2,5].

Females were older than males in this study. This has also been reported in Bangladesh (mean age 63.58 vs. 60.58 years) [35], Hong Kong (mean age 73.4 vs. 68.8 years) [36], Japan (median age 77 vs. 70 years) [37], Sri Lanka (median age 63 vs. 66 years) [38], Vietnam (mean age 67.7 vs. 63.7 years) [39]. It is consistent with the meta-analysis [5], and has been attributed to females living longer than males [2,3,5] and the increasing incidence of stroke with age [2].

There were sex differences in vascular risk factors in this study, and notably less SM among females compared to males. Differing patterns of sex differences in risk factors have been reported by various studies from Bangladesh [35], Malaysia [41], Sri Lanka [38] and Vietnam [39], but they have also consistently shown a lower frequency of SM. These are similar findings as the meta-analysis [8]. Of note is the excess risk of stroke associated with DM being significantly higher in females than males, independent of sex differences in other major cardiovascular risk factors [42]. With respect to SM, compared with non-smokers, the excess risk of stroke is at least as great among females who smoke compared with males who smoke [43], while elevated levels of systolic blood pressure have a broadly similar impact on cardiovascular outcomes in both sexes [44].

In this study, females had less haemorrhagic stroke than males. This is similar to other studies—Hong Kong (17.1% vs. 22.4%) [36], Sri Lanka (14.2% vs. 21.4%) [38] and Vietnam (21.8% vs. 24.3%) [39]; no difference was found in Bangladesh [35], and the converse in Japan (19.9 % vs. 19.5%) [37]. This study found that the lower frequency persisted even after adjusting for vascular risk factors including HPT. Females have contrarily a higher risk of ischaemic stroke—this study found they have a higher frequency of DM, a potent risk factor for ischemic stroke [45]. Other factors not measured in this study may also be involved.

Among haemorrhagic stroke patients in this study, females were older than males and had more HPT, but also more DM and less HL and SM. The age difference was also found in Japan [37], and was also seen in an earlier Singapore study [46]. The strongest risk factors for ICH include ageing, HPT, and SM, as well as ambient particulate matter pollution, solid fuel pollution of household air, and renal dysfunction [47]—these latter data were not collected in this study.

There were no sex differences in haemorrhagic stroke mechanisms in this study. A higher rate of ICH in men (21.3% vs. 15.6%) and higher rate of SAH in women (6.2% vs. 3.0%) was found in Vietnam (study n = 2300) [39]. In Japan [37], of ICH, 57.3% were in males with 42.7% were in females while for SAH it was 62.7% vs. 32.8% (study n = 183,080). The increased frequency of SAH in females compared to males has been reported in meta-analyses [5,48]. The lack of this finding in this study may be due to the smaller sample size (study n = 1165) compared to the larger Vietnam study (n = 2300) [39] and much larger Japan study (n = 183,080) [37]; in addition, the number and proportion of cases with SAH in this study is low (n = 15; 5.5%).

When only ischaemic stroke patients were analysed, again females were older than males, had more HL and less SM and pCeVD. In China, females were older than males [49]; this was also found in Malaysia [50]. In Taiwan, more SM and alcohol consumption were seen in males, while more IHD and obesity were seen in females [51]. Age was similar between females and males in India [52]. In Korea, females were older and had more HPT and HL but less SM than males [53].

This study did not show sex differences in IS mechanisms or syndromes, as was similarly reported in Sri Lanka [38]. In India, LAA was more common in males (21.3% vs. 14.8%), while CE secondary to rheumatic heart disease were more common in females (27.2% vs. 19.7%) [52]. Similarly, in Japan, males had more LAA (34.3% vs. 32.3%) while females had more CE (14.7% vs. 12.3%) [36]. Studies suggest that females have a high frequency of CE than males due to their higher frequency of atrial fibrillation [3]. A meta-analysis has shown that CE is proportionally more common in women, while LAA and SVO are proportionally more common in men [5]. The absence of sex differences seen in this study may be due to lack of statistical power from the small sample size (n = 1165, IS n = 891) compared to the study from Japan (n = 183,080, IS n = 135,266) [37]. The Sri Lanka study had similar sample as this study (n = 949, IS n = 774) and had similar findings [38]. The India study, while having a smaller sample size (IS n = 742), had a high proportion of rheumatic heart disease (27.2% among females and 19.7% among males) [52], a disease very rare in Singapore.

There are a number of possible mechanisms to explain the sex differences seen in this study. The first is differences in vascular risk factors between the females and males [8]. However, the possible confounding effects of age, HPT, DM, HL, and SM were evaluated in the study by including these factors in the multivariable regression analysis comparing females and males. Another possibility is the effect of factors not assessed in this study. These include differences in the adequacy of control or compliance with medications for vascular risk factors, obesity, dietary patterns, life-style practices including the use of illicit drugs, and frequency and intensity of physical exercise—these are limitations of this study. A third explanation could be hormonal, from the vascular protective effect in females with endogenous oestrogen [11]. Use of OCP or HRT by females in this study is unlikely to be the cause as only two females were taking them. Admittedly, menopausal status was not considered. There may also be other biological differences between females and males that were not studied here, as well as genetic differences.

Global disparities in stroke burden have been attributed to population structures, risk factor prevalence, detection and control, stroke and risk factors awareness, access to and affordability of healthcare including primary care and acute and longer-term stroke services, and a country’s or region’s socioeconomic status [1]. Ethnicity and genetic factors, particularly those with environmental interactions, may also have a part to play in stroke risk [54]. Ethnicity and cultural beliefs may impact stroke awareness [55], stroke symptoms [56], access to care and outcomes [57], and use of post-acute care services [58] including rehabilitation [59]. Even intervention outcomes may be impacted [60]. Interethnic differences in stroke in Asia have been poorly studied—this may be due to the fact that most Asian counties are largely populated by a single ethnicity. Prior studies in Singapore [30] and Malaysia [61], both of which have a substantial ethnic Indian populations (7.5% [26] and 6.4% [62], respectively), have found that fewer Indians were admitted for stroke than the other ethnicities. Ethnic differences in IS mechanism using TOAST criteria found in this study were also reported in another Singapore study [63]; the lack of ethnic differences in IS syndrome using OSCP classification in this study were also reported in another Singapore study [64]. However, neither of those studies performed regression analysis to adjust for confounders; neither included ICH/SAH. A novel finding in this study is the differences in ethnicity between females and males with stroke, with more Chinese but fewer Indians among females compared to males. Sex–ethnicity diversity has been reported in other countries. An Irish stroke unit study comparing 437 ethnic Irish with 44 originally not of Irish ethnicity found fewer females among those not of Irish ethnicity compared to ethnic Irish (25% vs. 42.6%) [65]. A community-based stroke incidence study in New Zealand (n = 1,119,192) revealed that females were older than males at time of stroke across all ethnicities studied—European, Maori, Pacific Islanders, and Asian— with the oldest being European females (77 years) and the youngest being Pacific and Asian males (59 years) [66]. A nation-wide register-based cohort study in the Netherlands (n = 7,423,174) showed that Moroccan men and women had lower (adjusted hazard ratio aHR 0.42, 95% CI 0.36–0.48; aHR 0.37, 95% CI 0.30–0.46) while Surinamese men and women had a higher (aHR 1.43, 95% CI 1.35–1.50; aHR 1.34, 95% CI 1.28–1.41, respectively) risk of stroke of all types and subtypes compared to ethnic Dutch; further Turkish women had a lower incidence of SAH while Turkish men had an increased incidence of IS and ICH compared with ethnic Dutch SAH [67]. In a hospital-based registry study in South Africa (n = 524), there were slightly fewer white females than white males (0.9:1) while the numbers were similar among Black patients; the oldest were white females (mean age 66 +/− 15 years) while the youngest were black males (mean age 50 +/− 17 years) [68]. In the population-based South London Stroke Register (n = 271,871) in the United Kingdom, of IS subtypes, the incidence in Black ethnic groups were significantly higher for SVO in both sexes, and for Others in Black females compared with whites [69]. In the Atherosclerosis Risk in Communities Study (ARIC) in the USA (n = 15,792), the age-adjusted incidence rate of total strokes was highest among Black men (4.44/1000 person years)), followed by Black women (3.10/1000 person-years), white men (1.78/1000 person-years), and white women (1.24/1000 person-years) [70]. Prior studies in Singapore [30] and Malaysia [61] had reported that fewer Indian males in Singapore were admitted for stroke compared to the other ethnicities. Identifying a sex–ethnic group at high risk would help healthcare authorities to focus particular attention on those individuals so as to reduce their risk of having or being treated for stroke.

There are a number of study limitations. This is a single-centre study and may not reflect fully the Singapore situation. It included modest numbers of patients, and may not have been large enough to detect some statistically significant differences due to under-powering. It is a retrospective study based on case records, which is prone to missing data. There is also the possibility of selection bias. Data were not available on adequacy of control or compliance with medications for vascular risk factors, obesity, dietary patterns, and life-style practices including the use of illicit drugs or the frequency or intensity of physical exercise. There were no data on depression. The menopausal status of the female patients was not analysed. Still there are some strengths. The sample size is not small as it exceeds 1000 subjects. Data were collected in a uniform way in a standardised format. The patients could be well-characterised based on the available data. The results are consistent with other publications. Novel findings on ethnicity were found which adds to the minimal literature on this topic, especially among Asians.

## 5. Conclusions

In conclusion, this study has shown sex differences between females and males admitted to hospital for acute stroke. The findings of older age, more diabetes mellitus, less smoking history, less haemorrhagic stroke among females compared to males are consistent with the published literature. An increased frequency of subarachnoid haemorrhage or cardioembolic stroke among females reported in other populations could not be found. Novel is that ethnic differences were found, with more Chinese and fewer Indians among females compared to males. The findings of this study need to be corroborated by multi-centre studies with larger patient numbers, to prospectively validate these sex and ethnic differences and to spur more research to better understand sex differences in stroke, especially in Asian and multi-ethnic populations.

## Figures and Tables

**Table 1:** Sex differences in treatments and outcomes after stroke.

Parameter	Finding	Comment	Ref
Receiving intravenous thrombolytic therapy for acute IS	Females at lower odds than males	Substantial inter-study variability	[13]
Outcomes after receiving intravenous thrombolytic therapy for acute IS	Females less likely to obtain good or excellent, and more likely to have poor, functional outcomes	No significant difference in the complications of symptomatic ICH	[14]
mRS 0–2 at 90 days after endovascular thrombectomy for acute IS	No difference between females and males	When outliers were removed, the rate of mRS 0–2 and mRS 0–1 was lower among females compared to males	[15]
Difference in functional outcome by binary or ordinal analysis of mRS after endovascular thrombectomy for acute IS	No difference between females and males		[16]
Case fatality rates among those with aneurysmal SAH	No difference between females and males		[17]
Mortality at 1 and 10 years; stroke recurrence favourable outcome at 1 year	Females had higher mortality; higher stroke recurrence and lower favourable outcome		[18]
Mortality after stroke	IS: females had lower risk of mortality in-hospital, at one month, 12 months and five years HS: females had higher mortality risk in-hospital with no gender differences after discharge		[19]
Return to work after stroke	Females less likely than males		[20]
Medication prescriptions	Females less likely to be prescribed anti-thrombotics or lipid-lowering drugs, but more likely to receive anti-hypertensive drugs		[21]
DOACs for atrial fibrillation	Females may have higher risk of gastrointestinal bleeding with rivaroxaban and possibly dabigatran compared to warfarin	Little data on apixaban and edoxaban	[22]
CEA and CAS among symptomatic patients	CEA: higher rate of strokes and deaths in females CAS: higher incidence of perioperative myocardial infarction, stroke and long-term mortality among females		[23]
Neuroprotectant trials for stroke	Uric acid, dexborneol: treatment benefit in females but not males Tirilazad: worse treatment outcomes in females, no effect in males		[24]
Nutritional supplementation for stroke recover	No sex difference in response to intensive nutritional supplements, high proteins or amino acids, vitamins B or C or D, calcium, magnesium		[25]

(Legend: CEA = carotid endarterectomy; CS = carotid artery stenting; DOACS = direct acting oral anticoagulants; HS = haemorrhagic stroke; ICH = intracerebral haemorrhage; IS = ischaemic stroke; mRS = modified Rankin score; SAH = subarachnoid haemorrhage).

**Table 2:** Patient demographics, vascular risk factors, and stroke subtype, by sex.

	Total (n = 1165)	Female (n = 552)	Male (n = 613)	*p*-Value	Adjusted Odds Ratio, (95% CI) (Model 1)	*p*-Value	Adjusted Odds Ratio, (95% CI) (Model 2)	*p*-Value
Mean age (SD) (years)	65.6 (12.9)	67.46 (12.85)	63.89 (12.91)	<0.001	1.03 (1.02–1.04)	<0.001	1.03	<0.001
Ethnicity (%) Chinese Malay Indian Others	83.0 8.8 7.4 0.8	86.4 8.3 4.9 0.4	79.9 9.3 9.6 1.1	0.005 0.002 0.55 0.002 0.17		0.001		<0.001
Hypertension (%)	63.4	65.0	62.0	0.30	1.10 (0.76–1.59)	0.61	1.13 (0.78–1.63)	0.53
Diabetes mellitus (%)	31.8	36.1	27.9	0.003	1.60 (1.11–2.30)	0.012	1.50 (1.04–2.17)	0.032
Hyperlipidaemia (%)	60.3	63.4	57.4	0.041	0.74 (0.47–1.15)	0.18	0.72 (0.46–1.12)	0.14
Smoking (%)	35.6	11.6	57.3	<0.001	0.09 (0.07–0.13)	<0.001	0.09 (0.06–0.12)	<0.001
Ischaemic heart disease (%)	16.8	18.5	15.3	0.16	1.23 (0.85–1.77)	0.27	1.20 (0.83–1.74)	0.32
Previous cerebrovascular events (%)	24.2	20.8	27.2	0.011	0.67 (0.49–0.93)	0.016	0.67 (48–0.92)	0.014
Stroke subtype—HS (%)	23.5	23.4	23.7	0.95			0.71 (0.51–0.98)	0.036

(Legend: CI = confidence interval; HS = haemorrhagic stroke; SD = standard deviation. Model 1—multivariable logistic regression of stroke risk factors. Model 2—multivariable logistic regression of stroke risk factors and stroke subtype).

**Table 3:** Patient demographics, vascular risk factors, and haemorrhagic stroke, by sex.

	Total (n = 274)	Female (n = 129)	Male (n = 145)	*p*-Value	Adjusted Odds Ratio, (95% CI) (Model 1)	*p*-Value	Adjusted Odds Ratio, (95% CI) (Model 2)	*p*-Value
Mean age (SD) (years)	64.7 (14.3)	66.4 (13.8)	63.2 (14.3)	0.062	1.05 (1.02–1.08)	<0.001	1.05 (1.03–1.09)	<0.001
Ethnicity (%) Chinese Malay Indian Others	86.8 9.9 2.6 0.7	90.6 7.8 0.8 0.8	83.3 11.8 4.2 0.7	0.21 0.54 0.27 0.08 0.92		0.19		0.14
Hypertension (%)	63.6	63.3	63.9	1.00	2.5 (1.14–5.56)	0.02	2.94 (1.30–6.67	0.009
Diabetes mellitus (%)	13.6	17.2	10.4	0.11	3.70 (1.47–9.09)	0.005	4.00 (1.57–10.0)	0.004
Hyperlipidaemia (%)	47.4	45.3	49.3	0.54	0.18 (0.07–0.46)	<0.001	0.15 (0.06–0.41)	<0.001
Smoking (%)	25.7	10.2	39.6	<0.001	0.13 (0.07–0.27)	<0.001	0.13 (0.06–0.27)	<0.001
Ischaemic heart disease (%)	11.8	10.9	12.5	0.71	0.84 (0.37–2.0)	0.72	0.80 (0.34–1.96)	0.65
Previous cerebrovascular events (%)	20.6	19.5	21.5	0.76	0.93 (0.47–1.86)	0.83	0.88 (0.44–1.75)	0.72
HS subtype (%) SAH ICH IVH	5.5 92.3 2.2	5.5 91.4 3.1	5.6 93.1 1.4	0.62				0.18

(Legend: CI = confidence interval; SD = standard deviation; HS = haemorrhagic stroke; ICH = intracerebral haemorrhage; IVH = intraventricular haemorrhage; SAH = subarachnoid haemorrhage. Model 1—sex and risk factors. Model 2—sex and risk factors and HS subtype).

**Table 4:** Patient demographics, vascular risk factors, and ischaemic stroke, by sex.

	Total (n = 891)	Female (n = 423)	Male (n = 468)	*p*-Value	Adjusted Odds Ratio, (95% CI) (Model 1)	*p*-Value	Adjusted Odds Ratio, (95% CI) (Model 2)	*p*-Value	Adjusted Odds Ratio, (95% CI) (Model 3)	*p*-Value
Mean age (SD) (years)	65.9 (12.5)	67.78 (12.2)	64.1 (12.5)	<0.001	1.03 (1.01–1.04)	<0.001	1.03 (1.01–1.04)	<0.001	1.03 (1.01–1.04)	<0.001
Ethnicity (%) Chinese Malay Indian Others	81.9 8.5 8.8 0.8	85.1 8.5 6.1 0.2	79.1 8.5 11.1 1.3	0.016 0.02 1.00 0.008 0.06		0.001		0.01		0.001
Hypertension (%)	63.4	65.7	61.3	0.19	0.93 (0.60–1.42)	0.72	0.91 (0.9–1.41)	0.68	0.93 (0.60–1.42)	0.73
Diabetes mellitus (%)	37.4	41.8	33.3	0.01	1.12 (0.74–1.71)	0.58	1.11 (0.73–1.70)	0.62	1.12 (0.74–1.71)	0.60
Hyperlipidaemia (%)	64.3	69.0	60.0	0.006	1.13 (0.67–1.92)	<0.001	0.14 (0.67–1.93)	0.64	1.14 (0.67–1.93)	0.64
Smoking (%)	38.7	12.1	62.8	<0.001	0.08 (0.05–0.11)	<0.001	0.08 (0.05–0.11)	<0.001	0.08 (0.05–0.11)	<0.001
Ischaemic heart disease (%)	18.4	20.8	16.2	0.08	1.33 (0.88–2.02)	0.17	1.36 (0.87–2.11)	0.65	1.33 (0.87–2.02)	0.18
Previous cerebrovascular events (%)	25.4	21.3	29.1	0.009	0.63 (0.43–0.91)	0.01	0.88 (0.44–1.75)	0.17	0.63 (0.43–0.91)	0.014
IS mechanism (%) SVO CE ATH Others Unknown	37.9 17.4 12.2 8.1 24.4	39.7 19.9 10.4 5.9 17.7	36.3 15.2 13.9 10.0 24.6	0.047				0.89		
IS syndrome (%) TACI PACI LACI POCI	26.7 9.0 55.1 9.2	29.1 8.0 54.1 8.7	24.6 9.8 56.0 9.6	0.42						0.81

(Legend: CI = confidence interval; SD = standard deviation; ATH = large artery atherosclerosis; CE = cardioembolism; SVO = small vessel occlusion; TACI = total anterior circulation infarction; PACI = partial anterior circulation infarction; LACI = lacunar infarction; POCI = posterior circulation infarction. Model 1—sex and risk factors. Model 2—sex and risk factors and IS mechanism. Model 3—sex and risk factors and IS syndrome).

## Data Availability

Data are available from the author on reasonable request.
